# 1344. Stakeholders’ Perspectives of the Establishment of SARS-CoV-2 Monoclonal Antibody Infusion Centers in Rural Southwestern Virginia: A Qualitative Analysis with Realist Interview Approach

**DOI:** 10.1093/ofid/ofad500.1181

**Published:** 2023-11-27

**Authors:** Hyun Sue Kim, Anthony Baffoe-Bonnie

**Affiliations:** Virginia Tech Carilion School of Medicine, Roanoke, Virginia; Carilion Clinic, Roanoke, Virginia

## Abstract

**Background:**

The COVID-19 pandemic prompted healthcare organizations around the country to rapidly set up infusion centers that were solely dedicated to delivering outpatient COVID-19 monoclonal (mAb) and antiviral infusions. We capture the process of setting up these mAb infusion centers from the perspectives of key stakeholders. These lessons learnt may inform or refine actions in future pandemics.

**Methods:**

A realist interview approach was undertaken to evaluate the effectiveness of the program development with ten key stakeholders and healthcare workers who were involved. In contrast to traditional interviewing methods where a straight question leads to a straight answer, realist interviewing involves capturing the interviewees’ experiences through conversations of teaching and learning. We interviewed ten key stakeholders and healthcare workers who were involved with developing the mAb infusion process from Dec 13th, 2021, to Jan 19th, 2022, over online videoconferencing.

**Results:**

A causal mechanism diagram using a context-mechanism outcome configuration was identified. There was a rapid development of multiple infusion sites over a short time frame with dynamic staffing and patient appointment models leading to rapid increases in mAb infusion capacity in southwestern Virginia.

**Figure 1.**

**Figure:**
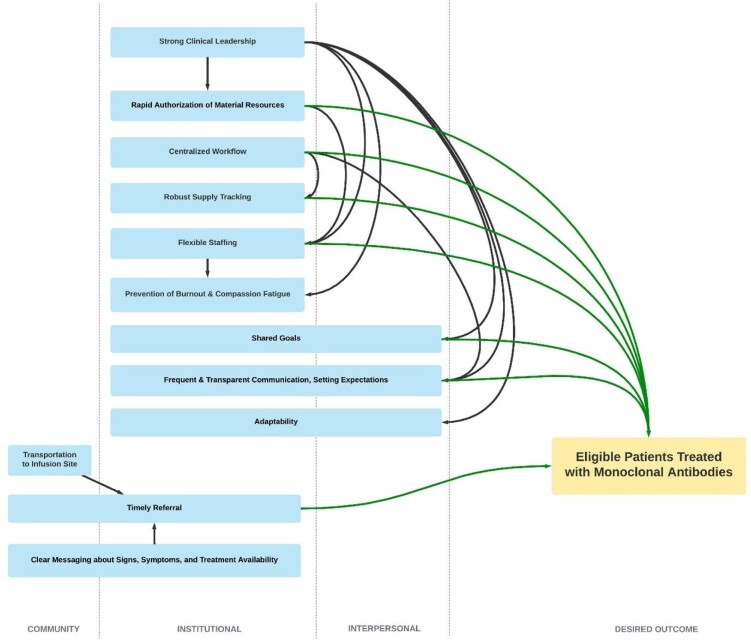

Causal mechanism diagram using a CMO configuration for the successful attributes leading to eligible patients treated with monoclonal antibodies.

**Conclusion:**

Strong clinical leadership, a robust communication and feedback framework and shared goals were identified by stakeholders as pivotal in the success of a COVID-19 monoclonal antibody infusion program for a health care system. Pandemic preparedness strategies should hardwire and leverage these features for resiliency.

**Disclosures:**

**All Authors**: No reported disclosures

